# Uses of 3-(2-Bromoacetyl)-2*H*-chromen-2-one in the Synthesis of Heterocyclic Compounds Incorporating Coumarin: Synthesis, Characterization and Cytotoxicity

**DOI:** 10.3390/molecules200611535

**Published:** 2015-06-23

**Authors:** Rafat M. Mohareb, Nadia Y. MegallyAbdo

**Affiliations:** 1 Chemistry Department, Faculty of Science, Cairo University, Giza 12613, Egypt; 2 Chemistry Department, Faculty of Education, Alexandria University, Alexandria 21526, Egypt; E-Mail: nadiamegally@yahoo.com

**Keywords:** coumarin, pyran, pyridine, thiazole, pyrazole, cytotoxicity

## Abstract

In this work, 3-bromoacetylcoumarin was used as the key starting material for the synthesis of pyran, pyridine, thiophene, thiazole and pyrazole derivatives through its reaction with different reagents. The structures of the newly synthesized compounds were confirmed on the basis of their spectral data and elemental analyses. All of the synthesized compounds were screened for their *in vitro* anticancer activity against six human cancer cell lines, namely: human gastric cancer (NUGC), human colon cancer (DLD1), human liver cancer (HA22T and HEPG2), nasopharyngeal carcinoma (HONE1), human breast cancer (MCF) and normal fibroblast cells (WI38). The IC_50_ values (the sample concentration that produces 50% reduction in cell growth) in nanomolars (nM)) showed most of the compounds exhibited significant cytotoxic effect. Among these derivatives, compound **6d** showed almost equipotent cytotoxic activity against NUGC (IC_50_ = 29 nM) compared to the standard CHS 828 (IC_50_ = 25 nM).

## 1. Introduction

Coumarins are a large group of naturally occurring compounds synthesized by numerous plant species as well as by some bacteria and fungi [1,2]. According to their chemical structure, they belong to the family of benzopyrones and represent a significant source of inspiration for new anticancer agents [3]. Benzopyran-2-ones are extremely variable in structure, due to various types of substitutions in their basic structure, which could influence their biological activity. A literature survey revealed their broad spectrum and diverse biological activities such as anti-microbial, anti-inflammatory, analgesic, anti-oxidant, antimalarial, anticancer, anti-tuberculosis and anti-HIV [4,5,6,7,8,9,10,11,12], particularly their cytotoxic activity against numerous types of cancers including malignan melanoma, leukemia, renal cell carcinoma, prostate and breast cancer cells progression [13,14,15]. Also, certain platinum (II) complexes of aminocoumarins show very good *in vitro* cytotoxicity [16]. A variety of mechanisms have been proposed, such as interfering with estrogen synthesis, interfering with cell cycle progression or even acting as inhibitors of cytochrome P450 1 [17].

**Figure 1:**
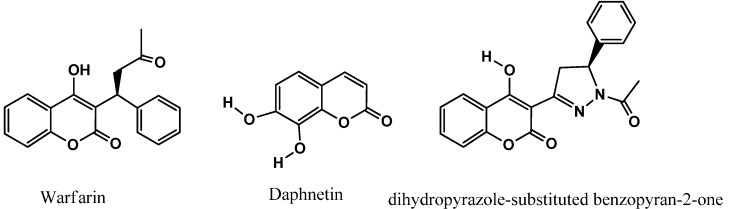
Anticancer and kinase inhibitors, benzopyrone derivatives.

Despite numerous attempts to search for more effective antitumor agents, coumarin still remains as one of the most versatile class of compound against cancer cell lines and are an important component among the molecules in drug discovery. Warfarin (Figure 1) reduces metastases from intestinal carcinomas to a great extent [18] and is also used as an adjunct to the surgical treatment of malignant tumors [19]. In addition, daphnetin (Figure 1) inhibits tyrosine kinase, epidermal growth factor receptor, serine/threonine- specific protein kinase, and protein kinase C *in vitro* [20]. Also, dihydropyrazole-substituted benzopyran-2-one (Figure 1) was identified as a novel class of MEK 1 kinase inhibitors [21].

Hybrid molecules, combining coumarins with different bioactive molecules like: pyran [22], pyridine [23], thiazole [24] and pyrazole [25] have recently been reported; these studies resulted in new compounds exhibiting significant anticancer activities.

On the basis of such findings, we report here the synthesis of new compounds containing the benzopyran-2-one nucleus substituted at position 3 with different bioisosteric moieties, such as pyran, pyridine, thiophene, thiazole and pyrazole, derivatives starting from the 3-(2-bromoacetyl)-2*H*-chromen-2-one (**1**) or 3-oxo-3-(2-oxo-2*H*-chromen-3-yl)propanenitrile (**8**). All of the newly synthesized compounds have been evaluated for their *in vitro* cytotoxicity against six human cancer cell lines and normal fibroblast cells.

## 2. Results and Discussion

### 2.1. Chemistry

In continuation of our work to synthesize polyfunctionalized biologically active heterocyclic compounds [26,27,28,29], we investigated the use of the 3-(2-bromoacetyl)-2*H*-chromen-2-one (**1**) [30,31] to synthesize thiophene, thiazole, pyrazole, pyran and pyridine derivatives incorporating a coumarin moiety. The aim of our work is the search for new possible anticancer agents. Thus, the reaction of compound **1** with benzenediazonium chloride gave the hydrazidic halide derivative **2**. The analytical and spectral data of compound **2** were the tools of its structure confirmation. Compound **1** reacted with malononitrile in the presence of ammonium acetate in an oil bath at 120 °C to give 2-(2-hydroxy-1-(2-oxo-2*H*-chromen-3-yl)ethylidene)malononitrile (**3**). This reaction involved an initial Knoevenagel condensation followed by hydrolysis of the α-bromo group into an OH moiety.

Next, we moved to studying the reactivity of compound **1** towards thiophene formation via the Gewald’s thiophene synthesis [32,33]. Thus, the reaction of compound **1** with elemental sulfur and either malononitrile or ethyl cyanoacetate in absolute ethanol solution containing triethylamine gave the thiophene derivatives **4a** and **4b**, respectively. The analytical and spectral data of the latter compounds were the basis of their structural elucidation. Thus, the ^1^H-NMR spectrum of compound **4a** (as an example) showed the presence of two singlets at δ 3.60, 6.90; corresponding to NH_2_ (D_2_O exchangeable) and coumarin H–4 in addition to a multiplet at δ 7.07–7.85; corresponding to the four aromatic protons. Moreover, the ^13^C-NMR spectrum showed the presence of δ 116.3 (CN), 166.2 (CO) along with the signals for coumarin and thiophene carbons.

**Scheme 1:**
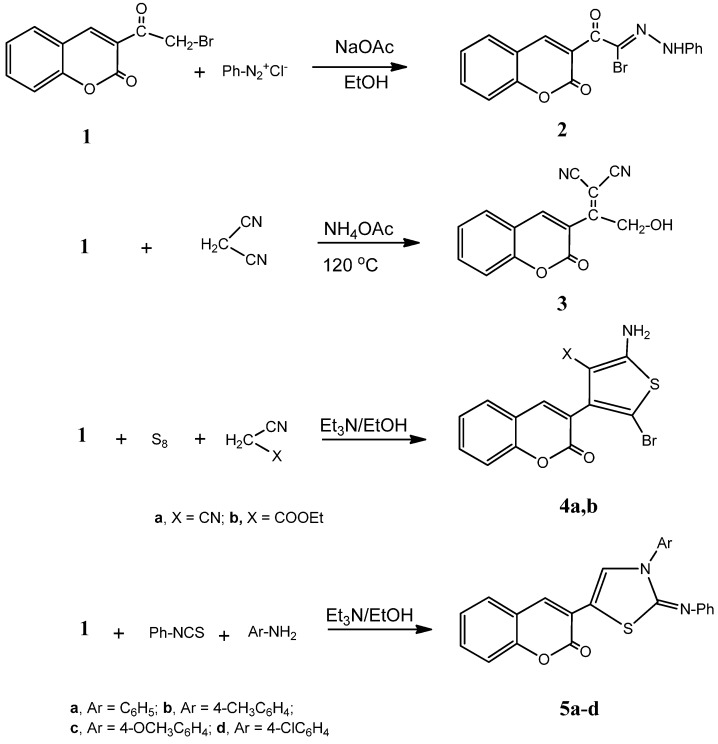
Synthesis of compounds **2**, **3**, **4a**, **b** and **5a**–**d**.

The presence of the α-bromocarbonyl moiety in compound **1** showed interesting reactivity towards thiazole formation. Thus, the reaction of compound **1** with phenylisothiocyanate and aromatic amines like either aniline, *p*-toluidine, 4-methoxyaniline or 4-chloroaniline gave the thiazole derivatives **5a**–**d**, respectively. The structures of the latter products were established on the basis of their respective analytical and spectral data. Thus, the ^1^H-NMR spectrum of **5a** showed the presence of two singlets at δ 3.99, 6.67 ppm corresponding to thiazole H–4 and coumarin H–4 in addition to a multiplet at δ 7.43–8.58; corresponding to 2C_6_H_5_ and C_6_H_4_ protons. In addition, the ^13^C-NMR spectrum revealed the presence of δ 164.3 (CO), 173.4 (C=N) beside the signals for coumarin, thiazole and 2C_6_H_5_ carbons (Scheme 1).

**Scheme 2:**
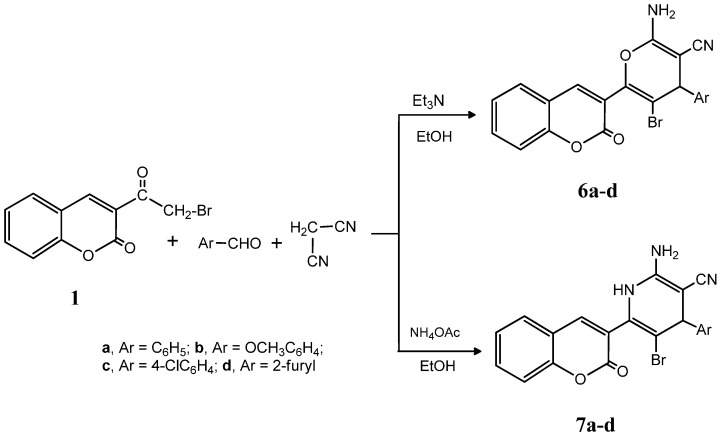
Synthesis of compounds **6a**–**d** and **7a**–**d**.

The multicomponent reactions of compound **1** with aromatic aldehydes and malononitrile were studied in order to generate potentially biologically active pyran and pyridine derivatives. Thus, the reaction of compound **1** with benzaldehyde, 4-methoxybenzaldehyde, 4-chlorobenzaldehyde or furfural gave the pyran derivatives **6a**–**d**, respectively. On the other hand, carrying the same reaction but using a catalytic amount of ammonium acetate instead of triethylamine gave the pyridine derivatives **7a**–**d**, respectively. The analytical and spectral data of **6a**–**d** and **7a**–**d** are consistent with their respective structures (see experimental section) (Scheme 2).

**Scheme 3:**
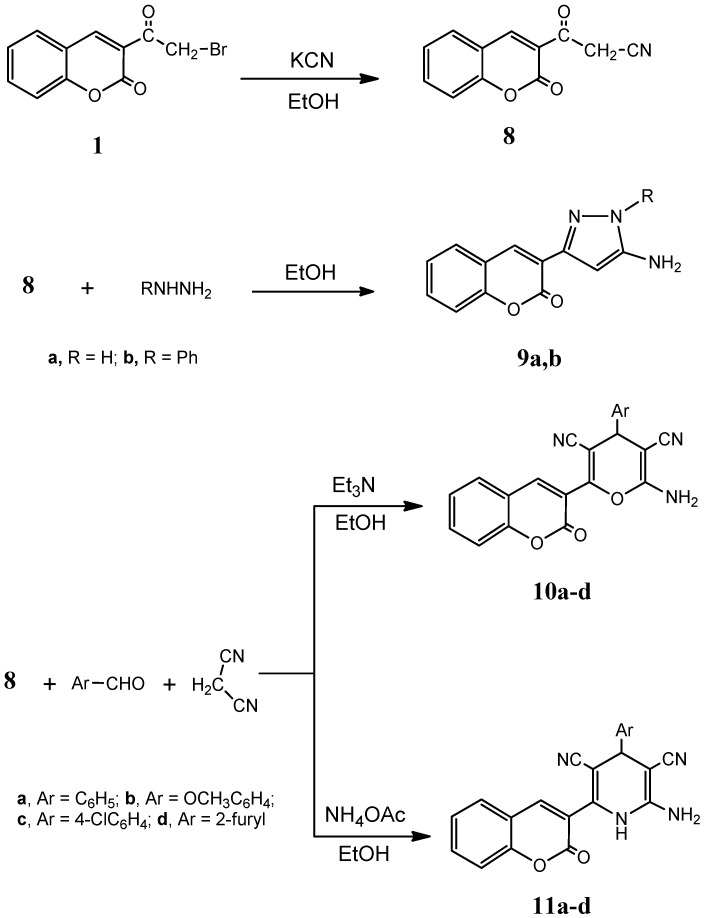
Synthesis of compounds **9a**, **b**, **10a**–**d** and **11a**–**d**.

The α-bromocarbonyl moiety present in compound **1** showed high reactivity towards nucleophilic displacement reactions. Thus, compound **1** reacted with potassium cyanide in aqueous medium to give the 3-oxo-3-(2-oxo-2*H*-chromen-3-yl)propanenitrile (**8**), the structure of which was based on analytical and spectral data. Compound **8** underwent heterocyclization reactions through its reaction with different chemical reagents. Thus, it reacted with either hydrazine hydrate or phenylhydrazine to give the pyrazole derivatives **9a** and **9b**, respectively. On the other hand, the multicomponent reaction of compound **8** with benzaldehyde, 4-methoxybenzaldehyde, 4-chlorobenzaldehyde or furfural gave the pyran derivatives **10a**–**d**, respectively. Alternatively, performing the same reaction but using a catalytic amount of ammonium acetate instead of triethylamine gave the pyridine derivatives **11a**–**d**, respectively (Scheme 3).The newly synthesized products were screened against different cancer cell lines where most of them showed remarkable activities.

### 2.2. In Vitro Cytotoxicity

#### 2.2.1. Effect on the Growth of Human Cancer Cell Lines

**Figure 2:**
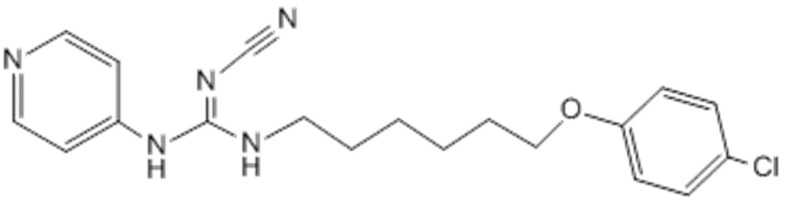
Chemical structure of CHS 828.

**Figure 3:**
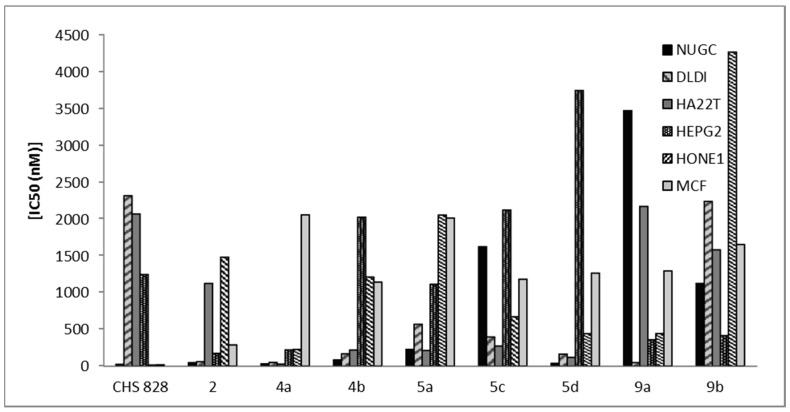
Cytotoxicity of compounds **2**, **4a**, **4b**, **5a**, **5c**, **5d**, **9a**, **9b** and CHS 828 against NUGC, gastric cancer; DLDI, colon cancer; HA22T, liver cancer; HEPG2, liver cancer; HONEI, nasopharyngeal carcinoma and MCF, breast cancer.

**Figure 4:**
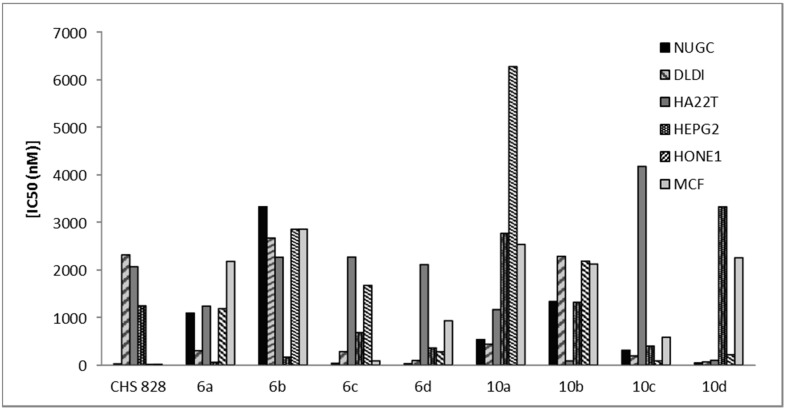
Cytotoxicity of 4*H*-pyran derivatives **6a**–**d**, **10a**–**d** and CHS 828 against NUGC, gastric cancer; DLDI, colon cancer; HA22T, liver cancer; HEPG2, liver cancer; HONEI, nasopharyngeal carcinoma and MCF, breast cancer.

**Figure 5:**
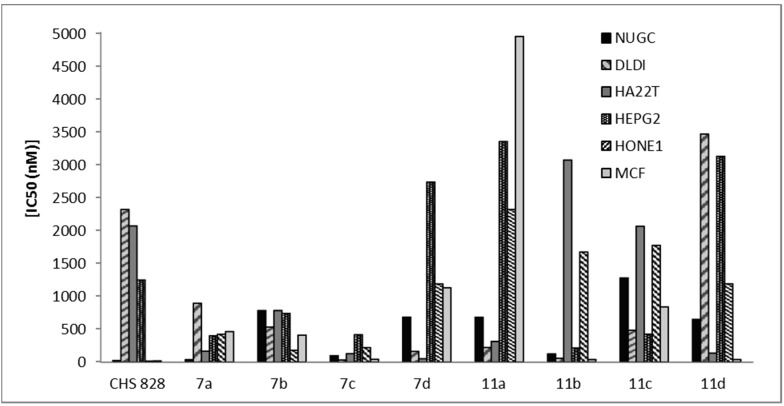
Cytotoxicity of 1,4-dihydropyridine derivatives **7a**–**d**, **11a**–**d** and CHS 828 against NUGC, gastric cancer; DLDI, colon cancer; HA22T, liver cancer; HEPG2, liver cancer; HONEI, nasopharyngeal carcinomaand MCF, breast cancer.

The heterocyclic compounds, prepared in this study, were evaluated according to standard protocols for their *in vitro* cytotoxicity against six human cancer cell lines including cells derived from human gastric cancer (NUGC), human colon cancer (DLD1), human liver cancer (HA22T and HEPG2), nasopharyngeal carcinoma (HONE1), human breast cancer (MCF) and normal fibroblast cells (WI38). For comparison purposes, CHS 828, a pyridyl cyanoguanidine, was used as a standard antitumor drug (Figure 2) [34]. All of the IC_50_ values (concentration that produces 50% reduction in cell growth) in nanomolars (nM) are listed in Table 1. All of the synthesized compounds showed potent inhibition with IC_50_ values in the nM range and the results are represented graphically in Figure 3, Figure 4 and Figure 5. All the synthesized compounds were tested for their cytotoxicity against normal fibroblast cells. The results obtained showed that normal fibroblast cells (WI38) were affected to a much lesser extent (IC_50_ > 10,000 nM).

**Table 1:** Cytotoxicity of compounds **2**, **3**, **4a**,**b**, **5a**–**d**, **6a**–**d**, **7a**–**d**, **9a**,**b**, **10a**–**d** and **11a**–**d** against a variety of cancer cell lines ^a^ [IC_50_^b^ (nM)].

Compound No.	Cytotoxicity (IC50 in nM)						
	NUGC	DLDI	HA22T	HEPG2	HONE1	MCF	WI38
**2**	48	60	1124	174	1480	288	na
**3**	1156	1280	1650	1226	699	821	910
**4a**	32	50	27	221	228	2055	780
**4b**	84	167	219	2023	1210	1142	na
**5a**	228	569	213	1112	2052	2011	632
**5b**	2211	1070	1288	1302	2179	1229	489
**5c**	1622	396	274	2120	670	1180	490
**5d**	38	163	120	3744	441	1264	860
**6a**	1092	303	1238	59	1185	2176	na
**6b**	3324	2667	2265	169	2853	2854	280
**6c**	38	283	2268	683	1672	89	480
**6d**	29	98	2109	360	279	931	na
**7a**	38	893	166	399	423	463	379
**7b**	782	532	783	738	180	409	160
**7c**	98	32	128	416	221	43	na
**7d**	682	163	52	2732	1186	1128	na
**9a**	3470	48	2169	359	442	1293	na
**9b**	1123	2237	1580	415	4266	1652	na
**10a**	537	440	1165	2766	6273	2533	417
**10b**	1335	2283	89	1320	2182	2121	na
**10c**	312	193	4173	399	89	584	na
**10d**	47	68	102	3322	220	2254	na
**11a**	680	222	314	3346	2316	4940	128
**11b**	124	58	3065	215	1670	39	na
**11c**	1277	483	2061	424	1770	839	na
**11d**	649	3460	137	3121	1188	40	652
**CHS 828**	25	2315	2067	1245	15	18	na

^a^ NUGC, gastric cancer; DLDI, colon cancer; HA22T, liver cancer; HEPG2, liver cancer; HONEI, nasopharyngeal carcinoma; MCF, breast cancer; WI38, normal fibroblast cells. ^b^ The sample concentration produces a 50% reduction in cell growth. na, not applicable.

#### 2.2.2. Structure Activity Relationship

In this study, when correlating the structures of the synthesized compounds with their anticancer activity, it has been observed that most of the synthesized compounds exhibited significant cytotoxic effects with IC_50_ values < 900 nM. Normal fibroblast cells (WI38) were affected to a much lesser extent (IC_50_ > 10,000 nM).

Phenylacetohydrazonoyl bromide derivative **2** was active against four cancer cell lines, namely NUGC, DLDI, HEPG2 and MCF with IC_50_ of 48, 60, 174 and 288 nM, respectively.

Comparing the cytotoxicity of the thiophene derivatives **4a** and **4b**, one can say that the cytotoxicity of **4a** was higher than that of **4b**. The presence of CN group with the thiophene ring in **4a** was responsible for its high potency.

Among the thiazole derivatives **5a**–**d**, compound **5d** is the most active derivative. It showed high potency against NUGC, DLDI, HA22T and HONEL with IC_50_ of 38, 163, 120 and 441 nM, respectively. Such high potency of **5d** is due to the presence of the 4-chlorophenyl moiety with the thiazole ring. The presence of *p*-tolyl moiety in **5b** decreases the activity relative to the unsubstituted phenyl derivative **5a**. On the other hand, the introduction of 4-OCH_3_ group in **5c** revealed better cytotoxicity against DLDI and HONEL than **5a**.

Considering the bromo-4*H*-pyran derivatives **6a**–**d**, compounds **6c** and **6d** revealed higher cytotoxic activity than **6a** and **6b**, both of them were active against most cancer cell lines. Compound **6d** showed almost equipotent activity against NUGC (IC_50_ = 29 nM) compared with the standard CHS 828 (IC_50_ = 25 nM). At the same time, **6c** exhibited the highest cytotoxicity among the four derivatives against MCF with IC_50_ = 89 nM. The reason for the high cytotoxicity of compounds **6c** and **6d** was attributed to the presence of the 4-chlorophenyl and the furan moieties, respectively.

The 5-bromo-1,4-dihydropyridine derivatives **7a**–**d** showed optimal cytotoxic activity. Compounds **7a**, **7b** and **7c** exhibited cytotoxic activity towards the six cancer cell lines. Compound **7c** incorporating with the 4-chlorophenyl moiety showed the highest potency among the four compounds with IC_50_ of 32 and 43 nM against DLDI and MCF, respectively. In general, the presence of the 5-bromopyridine moiety in compounds **7a**–**c** was responsible for their high potency.

Comparing the cytotoxicity of the pyrazole derivatives **9a** and **9b**, it was clear that the cytotoxicity of **9a** was higher than that of **9b**. It was clear that the *N*-phenylpyrazolyl moiety in compound **9b** was responsible for its lower potency.

Among the 4*H*-pyran-3,5-dicarbonitrile **10a**–**d**, compounds **10c** and **10d** showed higher cytotoxicity than **10a** and **10b**. Such high potency was attributed to the presence of 4-chlorophenyl group in the case of compound **10c**, and the furan moiety in case of compound **10d**, together with the pyran ring.

Among the 1,4-dihydropyridine-3,5-dicarbonitrile derivatives **11a**–**d**, compound **11b** with the 4-methoxyphenyl moiety showed the highest activity among the four derivatives. Compound **11b** showed high potency against NUGC, DLDI, HEPG2 and MCF cell lines with IC_50_ of 124, 58, 215 and 39 nM, respectively.

Comparing the cytotoxicity of the bromo-4*H*-pyran derivatives **6a**–**d** and 4*H*-pyran-3,5-dicarbonitrile **10a**–**d**, it was obvious that the presence of bromine atom together with the furan moiety in **6d** was responsible for its higher cytotoxicity than **10d**. Also the presence of a bromine atom in the 1,4-dihydropyridine derivatives **7a**–**c** revealed higher cytotoxic activity than the 1,4-dihydropyridine-3,5-dicarbonitriles **11a**–**c** [35,36].

## 3. Experimental

### 3.1. Chemistry

All melting points were determined on a Stuart apparatus and the values given are uncorrected. IR spectra (KBr, cm^−1^) were determined on a Shimadzu IR 435 spectrophotometer (Faculty of Pharmacy, Cairo University, Egypt). ^1^H-NMR and ^13^C-NMR spectra were recorded on Bruker Ascend 400 MHz spectrophotometers (Microanalytical Unit, Faculty of Pharmacy, Cairo University, Egypt) using TMS as the internal standard. Chemical shift values were recorded in ppm on δ scale. The electron impact (EI) mass spectra were recorded on a Hewlett Packard 5988 spectrometer (Microanalysis Center, Cairo University, Egypt). Elemental analyses were carried out at the Microanalysis Center, Cairo University, Egypt; found values were within ±0.35% of the theoretical ones. The progress of the reactions was monitored using thin layer chromatography (TLC) sheets precoated with UV fluorescent silica gel Merck 60F 254 and were visualized using UV lamp. The 3-(2-bromoacetyl)-2*H*-chromen-2-one (**1**) [30,31] was obtained using the reported procedure by the reaction of 3-acetylcoumarin in chloroform solution with bromine together with continuous stirring.

#### 3.1.1. Synthesis of 2-oxo-2-(2-oxo-2*H*-chromen-3-yl)-*N'*-phenylacetohydrazonoylbromide (**2**)

To a cold solution of the 3-(2-bromoacetyl)-2*H*-chromen-2-one (**1**) (2.67 g, 0.01 mol) in ethanol (30 mL) containing sodium acetate (2.5 g), a cold solution of benzenediazonium chloride (0.01 mol) (prepared by the addition of sodium nitrite solution (0.7 g, 0.01 mol) to a cold solution of aniline (0.93 g, 0.01 mol) in concentrated hydrochloric acid (12 mL) with continuous stirring) was added while stirring. The reaction mixture was kept at room temperature for 1 h and the formed solid product was collected by filtration and crystallized from ethanol. Yield: 85%;m.p.: 88–90 °C; IR (KBr, cm^−1^): 3425 (NH), 3058 (CH, aromatic), 1726, 1695 (2C=O), 1601 (C=N); ^1^H-NMR (DMSO-*d*_6_): δ 6.81 (s, 1H, coumarin H-4), 6.94–8.13 (m, 9H, C_6_H_5_, C_6_H_4_), 10.41 (s, 1H, NH, D_2_O exchangeable); ^13^C-NMR (DMSO-*d*_6_): δ 116.0, 118.9, 119.0, 119.2, 122.6, 124.8, 126.7, 129.6, 132.3, 134.6, 142.0, 143.1 (coumarin, C_6_H_5_ C), 164.0, 164.2 (2C=O), 175.3 (C=N); MS: *m*/*z* (%) 371 (M^+^). *Anal*. Calcd. for C_17_H_11_BrN_2_O_3_: C, 55.01; H, 2.99; N, 7.55. Found: C, 55.32; H, 3.29; N, 7.33.

#### 3.1.2. Synthesis of 2-(2-Hydroxy-1-(2-oxo-2*H*-chromen-3-yl)ethylidene)malononitrile (**3**)

A mixture of **1** (2.67 g, 0.01 mol), malononitrile (0.66 g, 0.1 mol) and ammonium acetate (0.5 g)were heated in an oil bath at 120 °C for 1 h then left to cool. The reaction product was dissolved in ethanol, poured onto ice water and neutralized by hydrochloric acid. The solid product was precipitated, filtered, washed with water, and crystallized from ethanol. Yield: 75%; m.p.: 162–164 °C; IR (KBr, cm^−1^): 3432 (OH), 3089 (CH, aromatic), 2206 (CN), 1709 (C=O); ^1^H-NMR (DMSO-*d*_6_): δ 5.15 (s, 2H, CH_2_), 6.95 (s, 1H, coumarin H-4), 7.15–7.96 (m, 4H, C_6_H_4_), 10.58 (s, 1H, OH, D_2_O exchangeable); ^13^C-NMR (DMSO-*d*_6_): δ 61.1 (CH_2_), 98.6, 102.3 (C=C), 116.8, 117.4 (2CN), 121.3, 123.6, 124.2, 125.8, 126.8, 129.4, 130.2, 132.9 (coumarin C), 163.5 (CO); MS: *m*/*z* (%) 252 (M^+^). *Anal*. Calcd. for C_14_H_8_N_2_O_3_: C, 66.67; H, 3.20; N, 11.11. Found: C, 66.32; H, 3.09; N, 11.05.

#### 3.1.3. General Procedure for the Synthesis of **4a**,**b**

A mixture of **1** (2.67 g, 0.01 mol) in absolute ethanol (40 mL) containing triethylamine (1.0 mL) and elemental sulfur (0.32 g, 0.01 mol) and either malononitrile (0.66 g, 0.01 mol) or ethyl cyanoacetate (1.13 g, 0.01 mol) was heated under reflux for 2 h. The reaction mixture was left to cool to room temperature and the formed solid product was collected by filtration and crystallized from ethanol.

*2-Amino-5-bromo-4-(2-oxo-2H-chromen-3-yl)thiophene-3-carbonitrile* (**4a**). Yield: 71%; m.p.: 180–182 °C; IR (KBr, cm^−1^): 3427 (NH_2_), 3034 (CH, aromatic), 2209 (CN), 1703 (C=O); ^1^H-NMR (DMSO-*d*_6_): δ 3.60 (s, 2H, NH_2_, D_2_O exchangeable), 6.90 (s, 1H, coumarin H-4), 7.07–7.85 (m, 4H, C_6_H_4_); ^13^C-NMR (DMSO-*d*_6_): δ 116.3 (CN), 119.3, 122.5, 124.2, 126.8, 129.6, 130.2, 134.5, 138.0, 139.8, 140.2, 143.8, 154.2 (coumarin, thiophene C), 166.2 (CO); MS: *m*/*z* (%) 347 (M^+^). *Anal*. Calcd. for C_14_H_7_BrN_2_O_2_S: C, 48.43; H, 2.03; N, 8.07; S, 9.24. Found: C, 48.68; H, 2.29; N, 8.39; S, 9.03.

*Ethyl 2-amino-5-bromo-4-(2-oxo-2H-chromen-3-yl)thiophene-3-carboxylate* (**4b**). Yield: 61%; m.p.: 177–179 °C; IR (KBr, cm^−1^): 3438 (NH_2_), 3089 (CH, aromatic), 1720, 1705 (2C=O); ^1^H-NMR (DMSO-*d*_6_): δ 1.15 (t, 3H, *J* = 7.2 Hz, CH_2_–CH_3_), 3.11 (q, 2H, *J* = 7.2 Hz, CH_2_–CH_3_), 3.69 (s, 2H, NH_2_, D_2_O exchangeable), 6.95 (s, 1H, coumarin H-4), 7.35–7.51 (m, 4H, C_6_H_4_); ^13^C-NMR (DMSO-*d*_6_): δ 22.3 (ester CH_3_), 58.7 (ester CH_2_), 119.3, 121.3, 122.8, 123.5, 124.8, 126.9, 127.3, 129.5, 130.8, 132.5, 134.9, 144.2 (coumarin, thiophene C), 166.0, 166.4 (2CO); MS: *m/z* (%) 394 (M^+^). *Anal*. Calcd. for C_16_H_12_BrNO_4_S: C, 48.74; H, 3.07; N, 3.55; S, 8.13. Found: C, 48.88; H, 3.39; N, 3.88; S, 7.89.

#### 3.1.4. General Procedure for the Synthesis of **5a**–**d**

A mixture of **1** (2.67 g, 0.01 mol), phenylisothiocyante (0.01 mol) and either of aniline (0.35 g, 0.01 mol), *p*-toluidine (0.04 g, 0.01 mol), 4-methoxyaniline (0.46 g, 0.01 mol) or 4-chloroaniline (0.47 g, 0.01 mol) in absolute ethanol (40 mL) containing triethylamine (1.0 mL) was heated under reflux for 2 h, left to cool to room temperature, poured onto ice/water, and neutralized by hydrochloric acid. The precipitated solid was collected by filtration, washed with water and crystallized from ethanol.

*3-(3-Phenyl-2-(phenylimino)-2*,*3-dihydrothiazol-5-yl)-2H-chromen-2-one* (**5a**). Yield: 76%; m.p.: 158–160 °C; IR (KBr, cm^−1^): 3064 (CH, aromatic), 1723 (C=O), 1609 (C=N); ^1^H-NMR (DMSO-*d*_6_): δ 3.99 (s, 1H, thiazole H-4), 6.67 (s, 1H, coumarin H-4), 7.43–8.58 (m, 14H, 2C_6_H_5_, C_6_H_4_); ^13^C-NMR (DMSO-*d*_6_): δ 119.3, 120.8, 121.3, 122.6, 124.3, 124.8, 126.2, 127.0, 127.3, 128.1, 129.2, 130.2, 132.8, 133.2, 138.4, 140.3, 142.8, 144.5 (coumarin, thiazole, 2C_6_H_5_ C), 164.3 (CO), 173.4 (C=N); MS: *m*/*z* (%) 396 (M^+^). *Anal*. Calcd. for C_24_H_16_N_2_O_2_S: C, 72.71; H, 4.07; N, 7.07; S, 8.09. Found: C, 72.43; H, 4.09; N, 7.29; S, 8.39.

*3-(2-(Phenylimino)-3-(p-tolyl)-2*,*3-dihydrothiazol-5-yl)-2H-chromen-2-one* (**5b**). Yield: 69%; m.p.: 99–101 °C; IR (KBr, cm^−1^): 3033 (CH, aromatic), 1721 (C=O), 1600 (C=N); ^1^H-NMR (DMSO-*d*_6_): δ 2.25 (s, 3H, CH_3_), 3.98 (s, 1H, thiazole H-4), 6.60 (s, 1H, coumarin H-4), 7.09–7.50 (m, 13H, C_6_H_5_, 2C_6_H_4_); ^13^C-NMR (DMSO-*d*_6_): δ 20.8 (CH_3_), 120.2, 121.4, 121.8, 122.4, 123.9, 124.4, 125.2, 126.9, 128.0, 130.2, 132.5, 133.2, 136.3, 138.8, 141.6, 142.9, 143.4, 144.6 (coumarin, thiazole, C_6_H_5_, C_6_H_4_ C), 164.1 (CO), 173.8 (C=N); MS: *m*/*z* (%) 410 (M^+^). *Anal*. Calcd. for C_25_H_18_N_2_O_2_S: C, 73.15; H, 4.42; N, 6.82; S, 7.81. Found: C, 73.45; H, 4.09; N, 6.69; S, 7.64.

*3-(3-(4-Methoxyphenyl)-2-(phenylimino)-2*,*3-dihydrothiazol-5-yl)-2H-chromen-2-one* (**5c**). Yield: 72%; m.p.: 103–105 °C; IR (KBr, cm^−1^): 3053 (CH, aromatic), 1717 (C=O), 1603 (C=N); ^1^H-NMR (DMSO-*d*_6_): δ 3.79 (s, 3H, OCH_3_), 4.45 (s, 1H, thiazole H-4), 6.82 (s, 1H, coumarin H-4), 6.88–7.50 (m, 13H, C_6_H_5_, 2C_6_H_4_); ^13^C-NMR (DMSO-*d*_6_): δ 32.9 (OCH_3_), 120.4, 120.9, 121.3, 123.0, 123.6, 124.1, 125.3, 127.3, 128.6, 130.6, 132.8, 136.4, 138.4, 138.9, 139.5, 140.8, 143.6, 144.8 (coumarin, thiazole, C_6_H_5_, C_6_H_4_C), 164.6 (CO), 173.2 (C=N); MS: *m*/*z* (%) 426 (M^+^). *Anal.* Calcd. for C_25_H_18_N_2_O_3_S: C, 70.40; H, 4.25; N, 6.57; S, 7.52. Found: C, 70.13; H, 4.08; N, 6.82; S, 7.29.

*3-(3-(4-Chlorophenyl)-2-(phenylimino)-2*,*3-dihydrothiazol-5-yl)-2H-chromen-2-one* (**5d**). Yield: 71%; m.p.: 123–125 °C; IR (KBr; cm^−1^): 3030 (CH; aromatic); 1719 (C=O); 1597 (C=N); ^1^H-NMR (DMSO-*d*_6_): δ 3.98 (s, 1H, thiazole H-4); 6.56 (s, 1H, coumarin H-4); 6.99–7.54 (m; 13H, C_6_H_5_, 2 C_6_H_4_); ^13^C-NMR (DMSO-*d*_6_): δ 119.8, 120.4, 121.2, 122.4, 123.9, 124.6, 125.4, 126.0, 127.6, 128.2, 129.1, 130.3, 131.2, 132.8, 137.3, 140.5, 142.8, 144.4 (coumarin, thiazole, C_6_H_5_, C_6_H_4_ C); 164.9 (CO), 173.5 (C=N); MS: *m*/*z* (%) 430 (M^+^). *Anal.* Calcd. for C_24_H_15_ClN_2_O_2_S: C; 66.90; H; 3.51; N; 6.50; S; 7.44. Found: C; 66.66; H; 3.77; N; 6.82; S; 7.69.

#### 3.1.5. General Procedure for the Synthesis of Compounds **6a**–**d**

A mixture of compound **1** (2.67 g, 0.01 mol), malononitrile (0.66 g, 0.01 mol) and either benzaldehyde (1.06 g, 0.01 mol), 4-methoxybenzaldehyde (1.36 g, 0.01 mol), 4-chlorobenzaldehyde (1.27 g, 0.01 mol) or furfural (0.96 g, 0.01 mol) in absolute ethanol (40 mL) containing triethylamine (1.0 mL) was heated under reflux for 2 h, left to cool to room temperature, poured onto ice/water, and neutralized by hydrochloric acid. The precipitated solid was collected by filtration, washed with water and crystallized from ethanol.

*2-Amino-5-bromo-6-(2-oxo-2H-chromen-3-yl)-4-phenyl-4H-pyran-3-carbonitrile* (**6a**). Yield: 68%; m.p.: 140–142 °C; IR (KBr, cm^−1^): 3408 (NH_2_), 3063 (CH, aromatic), 2212 (CN), 1723 (C=O); ^1^H-NMR (DMSO-*d*_6_): δ 3.46 (s, 2H, NH_2_, D_2_O exchangeable), 5.01 (s, 1H, pyran H-4), 7.02 (s, 1H, coumarin H-4), 7.24–7.98 (m, 9H, C_6_H_5_, C_6_H_4_); ^13^C-NMR (DMSO-*d*_6_): δ 65.8 (pyran C-4), 116.8 (CN), 121.3, 121.8, 122.4, 122.8, 123.2, 124.7, 126.7, 127.8, 128.3, 129.6, 130.6, 131.8, 133.9, 140.8, 142.3, 143.9 (coumarin, pyran, C_6_H_5_ C), 164.9 (CO); MS: *m*/*z* (%) 421 (M^+^). *Anal.* Calcd. for C_21_H_13_BrN_2_O_3_: C, 59.88; H, 3.11; N, 6.65. Found: C, 59.58; H, 3.02; N, 6.39.

*2-Amino-5-bromo-4-(4-methoxyphenyl)-6-(2-oxo-2H-chromen-3-yl)-4H-pyran-3-carbonitrile* (**6b**). Yield: 65%; m.p.: 193–195 °C; IR (KBr, cm^−1^): 3415 (NH_2_), 3070 (CH, aromatic), 2219 (CN), 1720 (C=O); ^1^H-NMR (DMSO-*d*_6_): δ 3.11 (s, 3H, OCH_3_), 3.46 (s, 2H, NH_2_, D_2_O exchangeable), 5.68 (s, 1H, pyran H-4), 7.09 (s, 1H, coumarin H-4), 7.34–7.87 (m, 8H, 2C_6_H_4_); ^13^C-NMR (DMSO-*d*_6_): δ 34.8 (OCH_3_), 65.4 (pyran C-4), 116.8 (CN), 119.3, 121.3, 122.4, 122.8, 123.2, 124.7, 125.1, 125.8, 126.7, 127.8, 129.3, 130.6, 133.9, 140.8, 142.3, 143.9 (coumarin, pyran, C_6_H_4_ C), 164.9 (CO); MS: *m*/*z* (%) 451 (M^+^). *Anal*. Calcd. for C_22_H_15_BrN_2_O_4_: C, 58.55; H, 3.35; N, 6.21. Found: C, 58.66; H, 3.12; N, 5.91.

*2-Amino-5-bromo-4-(4-chlorophenyl)-6-(2-oxo-2H-chromen-3-yl)-4H-pyran-3-carbonitrile* (**6c**). Yield: 65%; m.p.: 178–180 °C; IR (KBr, cm^−1^): 3410 (NH_2_), 3067 (CH, aromatic), 2211 (CN), 1720 (C=O); ^1^H-NMR (DMSO-*d*_6_): δ 3.31 (s, 2H, NH_2_, D_2_O exchangeable), 5.73 (s, 1H, pyran H-4), 6.73 (s, 1H, coumarin H-4), 6.93–7.72 (m, 8H, 2C_6_H_4_); ^13^C-NMR (DMSO-*d*_6_): δ 65.8 (pyran C-4), 116.6 (CN), 119.3, 120.8, 121.6, 122.3, 123.6, 123.9, 125.3, 125.9, 126.8, 127.3, 130.9, 132.2, 138.9, 140.2, 142.6, 143.1 (coumarin, pyran, C_6_H_4_ C), 164.6 (CO); MS: *m*/*z* (%) 455 (M^+^). *Anal*. Calcd. for C_21_H_12_BrClN_2_O_3_: C, 55.35; H, 2.65; N, 6.15. Found: C, 55.21; H, 2.95; N, 5.93.

*2-Amino-5-bromo-4-(furan-2-yl)-6-(2-oxo-2H-chromen-3-yl)-4H-pyran-3-carbonitrile* (**6d**). Yield: 73%; m.p.: 148–150 °C; IR (KBr, cm^−1^): 3420 (NH_2_), 3048 (CH, aromatic), 2216 (CN), 1727 (C=O); ^1^H-NMR (DMSO-*d*_6_): δ 3.31 (s, 2H, NH_2_, D_2_O exchangeable), 5.80 (s, 1H, pyran H-4), 7.12 (s, 1H, coumarin H-4), 7.30–8.00 (m, 7H, C_6_H_4_, furan); ^13^C-NMR (DMSO-*d*_6_): δ 65.8 (pyran C-4), 116.4 (CN), 118.9, 121.8, 122.1, 122.7, 123.2, 124.2, 125.2, 126.0, 126.4, 128.4, 130.9, 134.5, 141.6, 140.6, 143.9, 148.2 (coumarin, pyran, furan C), 164.6 (CO); MS: *m*/*z* (%) 411 (M^+^). *Anal*. Calcd. for C_19_H_11_BrN_2_O_4_: C, 55.50; H, 2.70; N, 6.81. Found: C, 55.31; H, 3.01; N, 6.62.

#### 3.1.6. General Procedure for the Synthesis of **7a**–**d**

A mixture of compound **1** (2.67 g, 0.01 mol), malonanitrile (0.66 g, 0.01 mol) and either benzaldehyde (1.06 g, 0.01 mol), 4-methoxybenzaldehyde (1.36 g, 0.01 mol), 4-chlorobenzaldehyde (1.27 g, 0.01 mol) or furfural (0.96 g, 0.01 mol) in absolute ethanol (40 mL) containing ammonium acetate (0.5 g) was heated under reflux for 3 h, left to cool to room temperature, poured onto ice/water, and neutralized with hydrochloric acid. The precipitated solid was collected by filtration, washed with water and crystallized from ethanol.

*2-Amino-5-bromo-6-(2-oxo-2H-chromen-3-yl)-4-phenyl-1*,*4-dihydropyridine-3-carbonitrile* (**7a**). Yield: 80%; m.p.: 171–173 °C; IR (KBr, cm^−1^): 3415–3346 (NH_2_, NH), 3064 (CH, aromatic), 2209 (CN), 1714 (C=O); ^1^H-NMR (DMSO-*d*_6_): δ 3.48 (s, 2H, NH_2_, D_2_O exchangeable), 7.15 (s, 1H, pyridine H-4), 7.20 (s, 1H, coumarin H-4), 7.34–7.69 (m, 9H, C_6_H_5_, C_6_H_4_), 9.16 (s, 1H, NH, D_2_O exchangeable); ^13^C-NMR (DMSO-*d*_6_): δ 62.3 (pyridine C-4), 116.9 (CN), 119.3, 120.3, 121.9, 123.8, 124.5, 124.8, 125.8, 126.2, 126.9, 128.0, 128.3, 130.3, 132.4, 139.3, 140.9, 143.2 (coumarin, pyridine, C_6_H_5_ C), 165.3 (CO); MS: *m*/*z* (%) 420 (M^+^). *Anal*. Calcd. for C_21_H_14_BrN_3_O_2_: C, 60.02; H, 3.36; N, 10.00. Found: C, 59.89; H, 3.18; N, 9.73.

*2-Amino-5-bromo-4-(4-methoxyphenyl)-6-(2-oxo-2H-chromen-3-yl)-1*,*4-dihydropyridine-3-carbonitrile* (**7b**). Yield: 82%; m.p.: 164–166 °C; IR (KBr, cm^−1^): 3407–3365 (NH_2_, NH), 3064 (CH, aromatic), 2207 (CN), 1710 (C=O); ^1^H-NMR (DMSO-*d*_6_): δ 3.66 (s, 3H, OCH_3_), 3.84 (s, 2H, NH_2_, D_2_O exchangeable), 6.92 (s, 1H, pyridine H-4), 6.95 (s, 1H, coumarin H-4), 6.97–7.98 (m, 8H, 2C_6_H_4_), 9.86 (s, 1H, NH, D_2_O exchangeable); ^13^C-NMR (DMSO-*d*_6_): δ 38.9 (OCH_3_), 62.8 (pyridine C-4), 116.3 (CN), 120.1, 120.3, 122.6, 123.2, 124.5, 124.6, 125.8, 126.8, 127.4, 129.8, 132.6, 136.2, 137.4, 139.2, 140.6, 143.8 (coumarin, pyridine, C_6_H_4_ C), 164.4 (CO); MS: *m*/*z* (%) 450 (M^+^). *Anal*. Calcd. for C_22_H_16_BrN_3_O_3_: C, 58.68; H, 3.58; N, 9.33. Found: C, 58.38; H, 3.28; N, 9.67.

*2-Amino-5-bromo-4-(4-chlorophenyl)-6-(2-oxo-2H-chromen-3-yl)-1*,*4-dihydropyridine-3-carbonitrile* (**7c**). Yield: 81%; m.p.: 206–208 °C;IR (KBr, cm^−1^): 3412–3360 (NH_2_, NH), 3055 (CH, aromatic), 2183 (CN), 1704 (C=O); ^1^H-NMR (DMSO-*d*_6_): δ 3.88 (s, 2H, NH_2_, D_2_O exchangeable), 6.92 (s, 1H, pyridine H-4), 6.95 (s, 1H, coumarin H-4), 6.98–7.94 (m, 8H, 2C_6_H_4_), 10.00 (s, 1H, NH, D_2_O exchangeable); ^13^C-NMR (DMSO-*d*_6_): δ 62.8 (pyridine C-4), 116.8 (CN), 120.4, 121.8, 122.9, 123.1, 124.3, 125.4, 126.9, 127.5, 128.3, 130.5, 133.2, 135.4, 136.8, 139.7, 140.2, 142.5 (coumarin, pyridine, C_6_H_4_ C), 164.8 (CO); MS: *m/z* (%) 454 (M^+^). *Anal*. Calcd. for C_21_H_13_BrClN_3_O_2_: C, 55.47; H, 2.88; N, 9.24. Found: C, 55.19; H, 3.08; N, 9.05.

*2-Amino-5-bromo-4-(furan-2-yl)-6-(2-oxo-2H-chromen-3-yl)-1*,*4-dihydropyridine-3-carbonitrile* (**7d**). Yield: 83%; m.p.: 205–207 °C; IR (KBr, cm^−1^): 3425–3387 (NH_2_, NH), 3045 (CH, aromatic), 2210 (CN), 1709 (C=O); ^1^H-NMR (DMSO-*d*_6_): δ 3.92 (s, 2H, NH_2_, D_2_O exchangeable), 6.46 (s, 1H, pyridine H-4), 6.77 (s, 1H, coumarin H-4), 6.90–8.08 (m, 7H, C_6_H_4_, furan), 8.82 (s, 1H, NH, D_2_O exchangeable); ^13^C-NMR (DMSO-*d*_6_): δ 63.0 (pryidine C-4), 116.9 (CN), 120.8, 121.3, 122.7, 123.5, 124.8, 125.6, 126.6, 127.2, 128.1, 129.2, 129.6, 130.6, 133.3, 135.3, 138.9, 144.7 (coumarin, pyridine, furan C), 166.2 (CO); MS: *m*/*z* (%) 410 (M^+^). *Anal*. Calcd. for C_19_H_12_BrN_3_O_3_: C, 55.63; H, 2.95; N, 10.24. Found: C, 55.39; H, 3.11; N, 10.51.

#### 3.1.7. Synthesis of 3-oxo-3-(2-oxo-2*H*-chromen-3-yl)propanenitrile (**8**)

A solution of compound **1** (2.67 g, 0.01 mol) in absolute ethanol (40 mL) was heated at 60 °C, then added to a solution of KCN (0.65 g, 0.01 mol in 10 mL water). The mixture was stirred for 0.5 h and the product was precipitated by adding ice and few drops of hydrochloric acid. The precipitated solid was collected by filtration, washed with water and crystallized from ethanol. Yield: 85%; m.p.: 158–160 °C; IR (KBr, cm^−1^): 3091 (CH, aromatic), 2247 (CN), 1739 (C=O); ^1^H-NMR (DMSO-*d*_6_): δ 5.08 (s, 2H, CH_2_), 6.63 (s, 1H, coumarin H-4), 6.88–7.83 (m, 4H, C_6_H_4_); ^13^C-NMR (DMSO-*d*_6_): δ 61.1 (CH_2_), 116.3 (CN), 121.0, 122.6, 123.8, 125.0, 126.2, 127.2, 129.4, 130.3, 133.2 (coumarin C), 162.2 (CO). MS: *m*/*z* (%) 213 (M^+^). *Anal*. Calcd. for C_12_H_7_NO_3_: C, 67.61; H, 3.31; N, 6.57. Found: C, 67.35; H, 3.11; N, 6.78.

#### 3.1.8. General Procedure for the Synthesis of Compounds **9a**,**b**

A solution of compound **8** (2.13 g, 0.01 mol) and either hydrazine hydrate (0.5 g, 0.01 mol) or phenylhydrazine (1.08 g, 0.01 mol) in absolute ethanol (40 mL) was heated under reflux for 2 h, left to cool to room temperature, poured onto ice/water containing few drops hydrochloric acid. The resulting product was collected by filtration, washed with water and crystallized from ethanol.

*3-(5-Amino-1H-pyrazol-3-yl)-2H-chromen-2-one* (**9a**). Yield: 83%;m.p.: 218–220 °C; IR (KBr, cm^−1^): 3416–3368 (NH_2_, NH), 3044(CH, aromatic), 1718 (C=O), 1611 (C=N); ^1^H-NMR (DMSO-*d*_6_): δ 3.92 (s, 2H, NH_2_, D_2_O exchangeable), 6.81 (s, 1H, pyrazole H-4), 6.90 (s, 1H, coumarin H-4), 6.93–7.84 (m, 4H, C_6_H_4_), 11.19 (s, 1H, NH, D_2_O exchangeable); ^13^C-NMR (DMSO-*d*_6_): δ 121.0, 122.6, 124.2, 125.9, 129.0, 130.6, 133.3, 135.3, 138.9, 140.2 (coumarin, pyrazole C), 165.3 (CO), 172.6 (C=N); MS: *m*/*z* (%) 227 (M^+^). *Anal*. Calcd. for C_12_H_9_N_3_O_2_: C, 63.43; H, 3.99; N, 18.49. Found: C, 63.52; H, 4.25; N, 18.22.

*3-(5-Amino-1-phenyl-1H-pyrazol-3-yl)-2H-chromen-2-one* (**9b**) Yield: 85%; m.p.: 158–160 °C; IR (KBr, cm^−1^): 3430 (NH_2_), 3056 (CH, aromatic), 1721 (C=O), 1607 (C=N); ^1^H-NMR (DMSO-*d*_6_): δ 3.88 (s, 2H, NH_2_, D_2_O exchangeable), 6.85 (s, 1H, pyrazole H-4), 6.89 (s, 1H, coumarin H-4), 6.95–7.83 (m, 9H, C_6_H_5_, C_6_H_4_); ^13^C-NMR (DMSO-*d*_6_): δ 120.3, 121.3, 122.9, 123.5, 124.8, 126.4, 127.4, 130.8, 131.4, 133.2, 135.6, 133.1, 136.5, 138.0 (coumarin, pyrazole, C_6_H_5_ C), 165.8 (CO), 172.3 (C=N); MS: *m*/*z* (%) 303 (M^+^). *Anal*. Calcd. for C_18_H_13_N_3_O_2_: C, 71.28; H, 4.32; N, 13.85. Found: C, 71.53; H, 4.09; N, 13.92.

#### 3.1.9. General Procedure for the Synthesis of Compounds **10a**–**d**

A mixture of compound **8** (2.67 g, 0.01 mol), malonanitrile (0.66 g, 0.01 mol) and either benzaldehyde (1.06 g, 0.01 mol), 4-methoxybenzaldehyde (1.36 g, 0.01 mol), 4-chlorobenzaldehyde (1.27 g, 0.01 mol) or furfural (0.96 g, 0.01 mol) in absolute ethanol (40 mL) containing triethylamine (1.0 mL) was heated under reflux for 2 h, left to cool to room temperature, poured onto ice/water, and neutralized by hydrochloric acid. The precipitated solid was collected by filtration, washed with water and crystallized from ethanol.

*2-Amino-6-(2-oxo-2H-chromen-3-yl)-4-phenyl-4H-pyran-3*,*5-dicarbonitrile* (**10a**). Yield: 88%; m.p.: 173–175 °C; IR (KBr, cm^−1^): 3432 (NH_2_), 3064 (CH, aromatic), 2200 (CN), 1723 (C=O); ^1^H-NMR (DMSO-*d*_6_): δ 3.73 (s, 2H, NH_2_, D_2_O exchangeable), 6.78 (s, 1H, pyran H-4), 6.85 (s, 1H, coumarin H-4), 7.14–7.92 (m, 9H, C_6_H_5_, C_6_H_4_); ^13^C-NMR (DMSO-*d*_6_): δ 62.8 (pyran C-4), 116.3, 117.3 (2CN), 119.8, 120.8, 123.2, 124.2, 125.1, 126.8, 127.9, 128.4, 129.3, 130.1, 132.3, 133.4, 134.8, 135.1, 138.2, 140.6 (coumarin, pyran, C_6_H_5_ C), 164.2 (CO); MS: *m*/*z* (%) 367 (M^+^). *Anal*. Calcd. for C_22_H_13_N_3_O_3_: C, 71.93; H, 3.57; N, 11.44. Found: C, 71.65; H, 3.88; N, 11.42.

*2-Amino-4-(4-methoxyphenyl)-6-(2-oxo-2H-chromen-3-yl)-4H-pyran-3*,*5-dicarbonitrile* (**10b**). Yield: 75%; m.p.: 113–115 °C; IR (KBr, cm^−1^): 3431 (NH_2_), 3053 (CH, aromatic), 2217 (CN), 1726 (C=O); ^1^H-NMR (DMSO-*d*_6_): δ 3.74 (s, 2H, NH_2_, D_2_O exchangeable), 3.88 (s, 3H, OCH_3_), 6.75 (s, 1H, pyran H-4), 6.89 (s, 1H, coumarin H-4), 6.95–7.99 (m, 8H, 2C_6_H_4_); ^13^C-NMR (DMSO-*d*_6_): δ 28.9 (OCH_3_), 63.1 (pyran C-4), 115.9, 116.2 (2CN), 118.3, 119.6, 121.8, 122.9, 123.5, 124.0, 125.3, 126.5, 127.2, 129.5, 130.4, 132.4, 134.2, 135.2, 136.8, 141.2 (coumarin, pyran, C_6_H_4_ C), 163.8 (CO). MS: *m*/*z* (%) 397 (M^+^). *Anal*. Calcd. for C_23_H_15_N_3_O_4_: C, 69.52; H, 3.80; N, 10.57. Found: C, 69.38; H, 3.62; N, 10.29.

*2-Amino-4-(4-chlorophenyl)-6-(2-oxo-2H-chromen-3-yl)-4H-pyran-3*,*5-dicarbonitrile* (**10c**). Yield: 88%; m.p.: 178–180 °C; IR (KBr, cm^−1^): 3432 (NH_2_), 3046 (CH, aromatic), 2198 (CN), 1725 (C=O); ^1^H-NMR (DMSO-*d*_6_): δ 3.89 (s, 2H, NH_2_, D_2_O exchangeable), 6.77 (s, 1H, pyran H-4), 6.89 (s, 1H, coumarin H-4), 6.98–8.05 (m, 8H, 2C_6_H_4_); ^13^C-NMR (DMSO-*d*_6_): δ 63.7 (pyran C-4), 116.2, 116.8 (2CN), 119.1, 119.6, 122.4, 123.1, 123.8, 124.7, 125.9, 127.3, 129.3, 131.1, 133.6, 134.8, 137.2, 138.2, 138.6, 141.8 (coumarin, pyran, C_6_H_4_ C), 164.9 (CO); MS: *m*/*z* (%) 401 (M^+^). *Anal*. Calcd. for C_22_H_12_ClN_3_O_3_: C, 65.76; H, 3.01; N, 10.46. Found: C, 65.42; H, 3.29; N, 10.72.

*2-Amino-4-(furan-2-yl)-6-(2-oxo-2H-chromen-3-yl)-4H-pyran-3*,*5-dicarbonitrile* (**10d**). Yield: 84%; m.p.: 133–135 °C; IR (KBr, cm^−1^): 3426 (NH_2_), 3033 (CH, aromatic), 2214 (CN), 1724 (C=O); ^1^H-NMR (DMSO-*d*_6_): δ 3.88 (s, 2H, NH_2_, D_2_O exchangeable), 6.51 (s, 1H, pyran H-4), 6.54 (s, 1H, coumarin H-4), 6.77–8.29 (m, 7H, C_6_H_4_, furan); ^13^C-NMR (DMSO-*d*_6_): δ 62.9 (pyran C-4), 116.4, 116.9 (2CN), 120.4, 122.4, 123.1, 123.8, 125.3, 127.3, 128.9, 129.4, 134.6, 136.2, 137.8, 138.6, 139.3, 140.1, 141.8, 144.8 (coumarin, pyran, furan C), 164.9 (CO); MS: *m*/*z* (%) 357 (M^+^). *Anal*. Calcd. for C_20_H_11_N_3_O_4_: C, 67.23; H, 3.10; N, 11.76. Found: C, 67.55; H, 2.86; N, 11.81.

#### 3.1.10. General Procedure for the Synthesis of Compounds **11a**–**d**

A mixture of compound **8** (2.67 g, 0.01 mol), malonanitrile (0.66 g, 0.01 mol) and either benzaldehyde (1.06 g, 0.01 mol), 4-methoxybenzaldehyde (1.36 g, 0.01 mol), 4-chlorobenzaldehyde (1.27 g, 0.01 mol) or furfural (0.96 g, 0.01 mol) in absolute ethanol (40 mL) containing ammonium acetate (0.5 g) was heated under reflux for 3 h, left to cool to room temperature, poured onto ice/water, and neutralized by hydrochloric acid. The precipitated solid was collected by filtration, washed with water and crystalized from ethanol.

*2-Amino-6-(2-oxo-2H-chromen-3-yl)-4-phenyl-1*,*4-dihydropyridine-3*,*5-dicarbonitrile* (**11a**) Yield: 85%; m.p.: 133–135 °C; IR (KBr, cm^−1^): 3430–3378 (NH_2_, NH), 3079 (CH, aromatic), 2197 (CN), 1716 (C=O); ^1^H-NMR (DMSO-*d*_6_): δ 3.88 (s, 2H, NH_2_, D_2_O exchangeable), 6.78 (s, 1H, pyridine H-4), 6.92 (s, 1H, coumarin H-4), 6.95–7.96 (m, 9H, C_6_H_5_, C_6_H_4_), 8.60 (s, 1H, NH, D_2_O exchangeable); ^13^C-NMR (DMSO-*d*_6_): δ 64.2 (pyridine C-4), 116.2, 116.8 (2CN), 119.1, 119.6, 120.8, 121.6, 122.4, 123.1, 123.8, 125.9, 127.3, 128.0, 129.2, 130.7, 133.6, 134.8, 137.2, 141.8 (coumarin, pyridine, C_6_H_5_ C), 164.3 (CO); (MS: *m*/*z* (%) 366 (M^+^). *Anal*. Calcd. for C_22_H_14_N_4_O_2_: C, 72.12; H, 3.85; N, 15.29. Found: C, 72.38; H, 4.13; N, 15.05.

*2-Amino-4-(4-methoxyphenyl)-6-(2-oxo-2H-chromen-3-yl)-1*,*4-dihydropyridine-3,5-dicarbonitrile* (**11b**). Yield: 90%; m.p.: 99–101 °C; IR (KBr, cm^−1^): 3429–3382 (NH_2_, NH), 3054 (CH, aromatic), 2221 (CN), 1720 (C=O); ^1^H-NMR (DMSO-*d*_6_): δ 3.75 (s, 3H, OCH_3_), 3.88 (s, 2H, NH_2_, D_2_O exchangeable), 6.77 (s, 1H, pyridine H-4), 6.87 (s, 1H, coumarin H-4), 6.90–7.99 (m, 8H, 2C_6_H_4_), 8.39 (s, 1H, NH, D_2_O exchangeable); ^13^C-NMR (DMSO-*d*_6_): δ 33.8 (OCH_3_), 63.8 (pyridine C-4), 116.0, 116.6 (2CN), 119.6, 120.8, 122.6, 122.9, 123.2, 124.6, 125.9, 126.2, 128.6, 128.8, 129.8, 130.9, 132.8, 134.3, 137.2, 144.2 (coumarin, pyridine, C_6_H_4_ C), 164.9 (CO); MS: *m*/*z* (%) 396 (M^+^). *Anal*. Calcd. for C_23_H_16_N_4_O_3_: C, 69.69; H, 4.07; N, 14.13. Found: C, 69.38; H, 4.09; N, 14.39.

*2-Amino-4-(4-chlorophenyl)-6-(2-oxo-2H-chromen-3-yl)-1*,*4-dihydropyridine-3,5-dicarbonitrile* (**11c**). Yield: 85%; m.p.: 197–199 °C; IR (KBr, cm^−1^): 3443–3375 (NH_2_, NH), 3054 (CH, aromatic), 2200 (CN), 1709 (C=O); ^1^H-NMR (DMSO-*d*_6_): δ 3.86 (s, 2H, NH_2_, D_2_O exchangeable), 6.74 (s, 1H, pyridine H-4), 6.96 (s, 1H, coumarin H-4), 7.09–7.97 (m, 8H, 2 C_6_H_4_), 10.00 (s, 1H, NH, D_2_O exchangeable); ^13^C-NMR (DMSO-*d*_6_): δ 63.9 (pyridine C-4), 116.2, 116.8 (2CN), 119.8, 120.3, 121.4, 122.6, 124.9, 125.2, 127.8, 129.3, 132.4, 133.0, 134.1, 137.2, 138.0, 139.3, 139.9, 144.0 (coumarin, pyridine, C_6_H_4_ C), 163.0 (CO); MS: *m*/*z* (%) 400 (M^+^). *Anal*. Calcd. for C_22_H_13_ClN_4_O_2_: C, 65.92; H, 3.27; N, 13.98. Found: C, 66.22; H, 3.02; N, 13.83.

*2-Amino-4-(furan-2-yl)-6-(2-oxo-2H-chromen-3-yl)-1*,*4-dihydropyridine-3,5-dicarbonitrile* (**11d**). Yield: 85%; m.p.: 168–170 °C; IR (KBr, cm^−1^): 3427–3375 (NH_2_, NH), 3034 (CH, aromatic), 2214 (CN), 1715 (C=O); ^1^H-NMR (DMSO-*d*_6_): δ 3.84 (s, 2H, NH_2_, D_2_O exchangeable), 6.55 (s, 1H, pyridine H-4), 6.90 (s, 1H, coumarin H-4), 6.98–8.09 (m, 7H, C_6_H_4_, furan), 8.81 (s, 1H, NH, D_2_O exchangeable); ^13^C-NMR (DMSO-*d*_6_): δ 63.8 (pyridine C-4), 116.3, 116.9 (2CN), 119.2, 120.7, 121.8, 122.3, 123.3, 126.9, 127.3, 128.3, 129.9, 130.6, 131.4, 132.8, 134.3, 136.4, 138.2, 143.4 (coumarin, pyridine, furan C), 164.8 (CO); MS: *m*/*z* (%) 356 (M^+^). *Anal*. Calcd. for C_20_H_12_N_4_O_3_: C, 67.41; H, 3.39; N, 15.72. Found: C, 67.66; H, 3.59; N, 15.88.

### 3.2. In Vitro Cytotoxic Assay

#### 3.2.1. Chemicals

Fetal bovine serum (FBS) and l-glutamine, were purchased from Gibco Invitrogen Co. (Scotland, UK). RPMI-1640 medium was purchased from Cambrex (East Rutherford, NJ, USA). Dimethyl sulfoxide (DMSO), CHS 828, penicillin, streptomycin and sulforhodamine B (SRB) were purchased from Sigma Chemical Co. (Saint Louis, MO, USA).

#### 3.2.2. Cell Cultures

Cell cultures were obtained from the European Collection of Cell Cultures (ECACC, Salisbury, UK) and human gastric cancer (NUGC), human colon cancer (DLD1), human liver cancer (HA22T and HEPG2), human breast cancer (MCF), nasopharyngeal carcinoma (HONE1) and normal fibroblast cells (WI38) were kindly provided by the National Cancer Institute (NCI, Cairo, Egypt). They were grown as a monolayer and routinely maintained in RPMI-1640 medium supplemented with 5% heat inactivated FBS, 2 mM glutamine and antibiotics (penicillin 100 U/mL, streptomycin 100 g/mL) at 37 °C in a humidified atmosphere containing 5% CO_2_. Exponentially growing cells were obtained by plating 1.5 × 10^5^ cells/mL for the six human cancer cell lines followed by 24 h of incubation. The effect of the vehicle solvent (DMSO) on the growth of these cell lines was evaluated in all the experiments by exposing untreated control cells to the maximum concentration (0.5%) of DMSO used in each assay.

## 4. Conclusions

The present study reports the successful synthesis, characterization and anticancer evaluation of new series of pyran, pyridine, thiophene, thiazole and pyrazole derivatives starting from 3-bromoacetylcoumarin through its reaction with different reagents. Most compounds showed potent inhibition with IC_50_ ˂ 900 nM. Among these derivatives, compound **6d** showed almost equipotent cytotoxic activity against NUGC (IC_50_ = 29 nM) compared to the standard CHS 828 (IC_50_ = 25 nM). Normal fibroblast cells (WI38) were affected to a much lesser extent (IC_50_ > 10,000 nM). The results suggest that these compounds may serve as lead chemical entities for further modification in the search of new classes of potential anticancer agents.
